# The Mediating Effect of Body Mass Index on the Association Between Problematic Social Media Use and Body Dysmorphic Concerns: A Cross‐Sectional Study Among Young Lebanese Adults

**DOI:** 10.1002/puh2.70316

**Published:** 2026-07-23

**Authors:** Carla Hokayem, Simona Mahfouz, Sahar Obeid, Michelle Abi Karam, Nada Akiki, Feten Fekih‐Romdhane, Souheil Hallit, Marie Hokayem

**Affiliations:** ^1^ School of Medicine and Medical Sciences Holy Spirit University of Kaslik Jounieh Lebanon; ^2^ Department of Nutrition and Food Sciences Faculty of Arts and Sciences Holy Spirit University of Kaslik Jounieh Lebanon; ^3^ Department of Psychology and Education School of Arts and Sciences Lebanese American University Jbeil Lebanon; ^4^ The Tunisian Center of Early Intervention in Psychosis Department of Psychiatry “Ibn Omrane” Razi Hospital Manouba Tunisia; ^5^ Faculty of Medicine of Tunis Tunis El Manar University Tunis Tunisia; ^6^ Applied Science Research Center Applied Science Private University Amman Jordan

**Keywords:** body dysmorphic concerns, body mass index, Lebanese, problematic social media use

## Abstract

**Background:**

The psychological mechanism that underlies the correlation between problematic social media use (PSMU) and body dysmorphic concerns was undetermined and ambiguous. The present study examined the mediating role of body mass index (BMI) in the association between PSMU and body dysmorphic concerns among a sample of Lebanese university students.

**Methods:**

A cross‐sectional study was conducted on 384 Lebanese university students, using an online survey collecting the following data: sociodemographic/socioeconomic characteristics, anthropometric measures, health status, dysmorphic concern, and PSMU.

**Results:**

In conducting the mediation analysis, adjustments were made for the following variables: sex, residence, age, household crowding index, and physical activity. BMI mediated the link between PSMU and body dysmorphic concerns (indirect effect: Beta = 0.03; BootSE = 0.01; 95% CI 0.01–0.07). Higher PSMU was significantly associated with higher BMI and directly linked to higher body dysmorphic concerns. Moreover, higher BMI was notably linked to higher body dysmorphic concerns.

**Conclusion:**

This study elucidates the connection between body dysmorphic concerns, PSMU, and BMI. Our findings suggest that BMI may partially explain the association between PSMU and body dysmorphic concerns among young Lebanese adults. Further longitudinal research is needed to better understand the mechanisms underlying these relationships. Studies conducted in different populations and different settings could assess if our results are local or generalizable.

## Graphical abstract

BMI partially mediates the association between problematic social media use and body dysmorphic concerns among Lebanese university students, with higher PSMU linked to increased BMI and greater dysmorphic concerns. These findings highlight BMI as a potential mechanism underlying the impact of social media on body image.

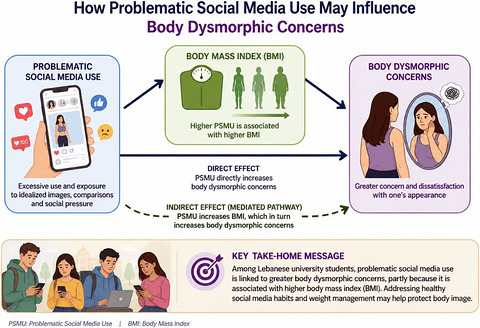

## 1 Introduction

Historically, considerable efforts were made to understand unusual forms of humans’ perception of their own body, irrespectively of how their body might actually look [1]. An over concern with a perceived flaw with one's appearance was seen as a distortion in the body image perception known as “body dysmorphic concern” (BDD) [2, 3]. Body dysmorphic concerns can range on a wide spectrum of severity, from mild and non‐impairing to clinically important [4]. In their severe forms, the concern in the perception of a slight “defect” in the physical appearance can cause major psychological distress and distortion in body's perception and, therefore, leads to the diagnosis of body dysmorphic disorder after ruling out other psychiatric and psychological conditions [2]. This condition was identified with a 1.7%–2.4% of prevalence in general population samples in Germany, Sweden, and the United States [5, 6]. Individuals with such concerns suffer from a preoccupation in this minor or even nonexistent defect, spending hours thinking about it, engaging in repetitive behaviors and mental acts, such as mirror gazing, appearance checking and comparison with others, with even trying to improve or conceal the body areas of concern [7, 8].

As for sociodemographic characteristics, “body dysmorphic concerns” showed variance inconsistency among genders. In some previous studies, males and females did not significantly differ, because although women were mainly preoccupied with their weight, their curves, and their skin, men showed comparable preoccupation with their physique, genitalia, and hair thinning [9]. Other studies indicated that females reported greater body dysmorphic concerns compared to men, knowing the existent social stereotypes and knowing that the female body and appearance were always being objectified and subjected to criticism and evaluation [10]. Furthermore, married individuals showed previously higher rates of body dysmorphic concerns compared to single individuals, potentially due to the partner's opinion that can be considered one of the social pressures [3]. However, early age of onset of body dysmorphic concern was linked to impaired functioning, well‐being, and quality of life [11]. In addition, residency in urban areas showed higher rates of body dysmorphic concerns compared to rural areas, and that might be attributed to the fact that body image and appearance were much more important to inhabitant of cities than rural and small villages [12]. Moreover, previous studies showed no association between body dysmorphic concerns and physical activity [13]; however, other studies demonstrated that greater physical activity showed less body dysmorphic concerns, and that might be explained by adequate level of physical exercise that helped in reducing body dissatisfaction [14]. Others linked physical activity to higher body dysmorphic concerns speaking of physical activity in this context as “compulsive exercises” or “exercise dependence” [15], influenced by many factors.

Previous research has explored the relationships between social media use, body mass index (BMI), and body image disturbances. These associations can be broadly categorized into three areas: the link between body dysmorphic concerns and problematic social media use (PSMU), the relationship between BMI and body image disturbances, and the potential association between PSMU and BMI.

### 1.1 Body Dysmorphic Concerns and PSMU

PSMU was one of the elements that was suggested as a risk factor for developing body dysmorphic concerns and potentially accounting for an increase even in their prevalence [16]. In fact, it is well‐known that social media has emerged as an integral part in people's live worldwide, for it transgressed connecting people, sharing posts, eliciting conversations, and uploading pictures [17]. Moreover, it was shown that social media contributes substantially to the development process of the human being, in shaping their identity, and in their perception of their body image [18]. Till now, no consensus was found on defining concepts related to PSMU, but constructs might include social media addiction, social media dependence, excessive use, or even problematic use of social media [19]. Nevertheless, research studies suggested that social PSMU can engender body dysmorphic concerns, due to multiple mechanisms, including self‐objectification and comparisons [18, 20]. Social media fosters an environment in which individuals feel compelled to seek approval for their appearance, which may engender an obsession over their hypothetical imperfections [21]. Therefore, this persistent exposure to idealized figures and standards may culminate in the establishment of body dysmorphic concerns and possibly exacerbating them [22].

### 1.2 BMI, Body Dysmorphic Concerns, and PSMU

BMI, which is the keystone of the present classification system for obesity, is generally employed to assess whether someone's weight falls below or above their ideal body weight [23]. It has been broadly utilized across domains, from worldwide surveillance to personal health assessment [24]. Some previous studies showed no relationship between BMI and the tendency of body dysmorphic concerns demonstrating that the latter can happen in any BMI [25], whereas others found that BMI was directly proportional to body dissatisfaction and thus body dysmorphic concerns [26]. Indeed, higher BMI was shown to foster an adverse self‐perception and thus influence deleteriously one's perceived body image leading to higher rates of body dysmorphic concerns [27]. Studies indicated that overweight individuals exhibited negative attitudes toward their own body [1, 28].

Other studies conversely linked BMI with PSMU, suggesting that individuals might spend time online looking for ideal body shapes and measurements, leading them to gain or lose appetite and weight in order to conform to the ideal norms and standards [29]. Higher PSMU was correlated with higher body dysmorphic concerns, which might be justified by the inappropriate PSMU that might lead to increased sitting time and thus a sedentary lifestyle [29, 30].

### 1.3 The Present Study

As mentioned previously, the associations, between PSMU and body dysmorphic disorder, BMI and PSMU, and BMI and body dysmorphic concerns, have been well established. However, till the day, the mediating effect of BMI between PSMU and body dysmorphic concerns remains largely unexplored. Investigating this pathway may help clarify possible mechanisms linking PSMU and body image disturbances among young adults. Therefore, knowing that the cultural background can sculpt and influence how the individual perceives his own body [31], and knowing that body dysmorphic concerns was thus a result of this culturally imposed paradigm [32]; it was interesting to conduct this study in the Middle East and specifically in Lebanon. In fact, it has been well established that in past decades, Western cultures introduced thin women and masculine men as role models, whereas Eastern cultures idealized women with curves, perceiving them as a symbol of fertility, health, and wealth; and masculinity as symbol of power [3]. Hence, appearance was always a main determinant of the manner individuals were judged in the society. When physical appearance influences the value ascribed to an individual, the desirability of physical attractiveness intensifies, thereby increasing the likelihood of developing body dysmorphic concerns [33]. Indeed, body dysmorphic concerns are most commonly found in teenagers and young adults irrespective of gender [34].

In fact, mental health disturbances often arise during youth (adolescence and young adulthood), which is a particularly vulnerable phase where a combination of environmental, psychological, and biological factors favor body dissatisfaction. Though the exact environmental determinants underlying the appearance and/or maintenance of body dysmorphic concerns remain to be pinpointed, social media use has been suggested as a powerful risk factor for body dysmorphic symptoms to the point of heightening the prevalence of BDD amongst youth [16, 35]. Accordingly, reducing social media use has been shown by Thai et al. [36] to be an effective method for inducing short‐term positive changes in body image within a vulnerable population of users (17–25 years) and, therefore, must be assessed as a potential element in the treatment of body‐image‐related disturbances. Moreover, another cross‐sectional study has demonstrated that increased social media use is associated with poorer body image in youth, a well‐established predictor of eating disorders [37]; with high‐quality experimental evidence being scarce and restricting the capacity to deduce robust causal interpretations.

Furthermore, in their cross‐sectional study, Saab et al. [38] found that 6.4% of university students with body dysmorphic concerns exhibited a higher propensity to develop anxiety, depression, and eating disorder with lower self‐esteem compared to those with no body dysmorphic concerns. In order to complete further the analysis of body dysmorphic concerns instigators in Lebanon, our study's aim was to assess the mediating effect of BMI between body dysmorphic concerns and PSMU among young Lebanese adults. Therefore, the primary hypothesis of this study was that higher BMI mediates the association between higher PSMU and higher body dysmorphic concerns among Lebanese university students.

## 2 Methods

### 2.1 Study Design and Participants

A cross‐sectional study was performed on Lebanese university students, aged 18–25 years old, in the districts of Keserwan and Jbeil, who consented to participate between March and May 2024. This study was reported in accordance with the Strengthening the Reporting of Observational Studies in Epidemiology (STROBE) guidelines [39]. The eligibility requirements for participants were as follows: studying at a university institution in Jbeil and Keserwan, being of Lebanese nationality aged between 18 and 25 years. Non‐Lebanese individuals, under psychotropic medications (antidepressants, antianxiety medications, stimulants, antipsychotics, and mood stabilizers), were excluded from the study. Participants were recruited using a non‐probability convenience sampling approach through online distribution of the questionnaire via social media platforms to collect data within 10‐min. All responses recorded were confidential and anonymous.

### 2.2 Minimal Sample Size

A minimal sample size of 123 was considered essential by means of the formula proposed by Fritz and MacKinnon [40] to determine the sample size: $=\frac{L}{f2}+k+1$, where *f* = 0.26 for medium effect size (based on Cohen's conventional benchmarks for regression‐based effect sizes), *L* = 7.85 for an *α* error of 5% and power *β* =  80%, and *k* = 6 variables to be entered in the model.

### 2.3 Questionnaire

This study utilized English and Arabic versions of a Google Form survey to collect data on sociodemographics, anthropometrics, body image, and patterns of social media use.

*Socio demographic data:* They consist of age, sex, marital status, residential area, and household crowding index (HCI). The HCI is defined by dividing the total household population (excluding newborns) by the number of rooms in the residence, omitting the kitchen [41]. *Anthropometric measurements* obtained were weight and height that were self‐reported by participants through the online questionnaire to assess the BMI (=weight in kg divided by square height in meters). Physical activity index was calculated by multiplying the intensity, duration, and frequency of the physical activity, with higher scores indicating higher physical activity [42].

*Social media disorder test (SMDT):* For screening PSMU, the SMDT uses just four items with a five‐level response format counting 1 = “never,” 2 = “rarely,” 3 = “sometimes,” 4 = “often,” and 5 = “very often.” The responses to the four questions can be summed to yield an overall score (ranging from 4 to 20), with higher scores indicating greater levels of PSMU as self‐assessed by the participants [43].

*Dysmorphic concern questionnaire (DCQ):* The DCQ assesses excessive concern with physical appearance using seven items rated on a 4‐point scale system where 0 represents “not at all,” 1 stands for “like most other people,” 2 denotes “more than other people,” and 3 signifies “much more than other people.” The DCQ items are summed to yield a single total score independent of subscales where greater scores relate to more disturbed aspects of one's physical appearance or functionality [44]. We used the Arabic version of the scale [45, 46].

### 2.4 Statistical Analyses

Data analysis was conducted via SPSS version 25. Reliability of the scales was assessed using Cronbach's alpha. The DCQ scores demonstrated a normal distribution, as indicated by the skewness (=0.264) and kurtosis (=0.029), both within the range of ±1. The Student *t*‐test was employed to compare continuous variables between two groups, whereas Pearson correlation was used to examine linear relationships between continuous variables. Correlation coefficients in terms of effect size were considered small for values <0.02, whereas values of 0.5 and 0.8 indicated medium and large effect, respectively [47]. Mediation analyses were performed using the PROCESS Macro version 3.4, model four, to evaluate three pathways [48]. Linearity assumptions were assessed by examining scatterplots between continuous predictors and outcomes, which indicated no substantial deviations from linearity. No evidence of problematic multicollinearity was found among variables included in the mediation model as verified by the variance inflation factor (VIF) values below 5. Pathway A identified the effect of the PSMU on BMI; Pathway B assessed the link between BMI and body dysmorphic concerns, and Pathways C and C′ estimated the total direct effect of PSMU on body dysmorphic concerns. The primary outcome variable was body dysmorphic concerns, measured using the DCQ. The primary independent variable was PSMU, assessed using the SMDT. BMI was examined as a mediating variable. Covariates included sex, age, marital status, residence, HCI, and physical activity. These variables were selected because previous studies have shown that sociodemographic and lifestyle factors may influence body image perceptions, BMI, and patterns of social media use [31]. Significance was determined when the Bootstrapped confidence interval excluded zero [26]. The mediation analysis was controlled for all variables with *p* < 0.25 in the bivariate analysis [49]. A *p* value of <0.05 was considered statistically significant.

## 3 Results

Three hundred eighty‐four Lebanese participants joined this study with a mean age of 21.28 years (SD = 1.94) and 53.9% males. Additional details regarding the sample are presented in Table 1.

**TABLE 1:** Sociodemographic characteristics of the participants (*n* = 384).

Variable	*n* (%)
Sex	
Male	207 (53.9)
Female	177 (46.1)
Marital status	
Single	368 (95.8)
Married	16 (4.2)
Residence	
Urban	221 (57.6)
Rural	163 (42.4)
Age (years)	21.28 ± 1.94
Household crowding index (person/room)	1.25 ± 0.43
Body mass index (kg/m^2^)	26.21 ± 4.65
Physical activity	15.93 ± 16.24
Problematic social media use	11.56 ± 3.51
Body dysmorphic concerns	7.88 ± 4.50

### 3.1 Bivariate Analysis

A higher mean body dysmorphic concern score was significantly present in males compared to females (8.45 ± 4.53 vs. 7.21 ± 4.38, *t*(382) = 2.72, *p* = 0.007) and in persons living in rural versus urban areas (8.64 ± 4.62 vs. 7.32 ± 4.33, *t*(382) = −2.87, *p* = 0.004). No significant difference was found between single and married people in terms of DCQ scores (7.93 ± 4.45 vs. 6.75 ± 5.50, *t*(382) = 1.02, *p* = 0.306). Greater PSMU and BMI were weakly‐to‐moderately linked to increased body dysmorphic concerns, whereas higher physical activity was significantly and weakly associated with lower body dysmorphic concerns (Table 2).

**TABLE 2:** Bivariate analysis of continuous variables associated with body dysmorphic concerns.

	1	2	3	4	5	6
1. Body dysmorphic concerns	1					
2. Problematic social media use	0.43***	1				
3. Physical activity	−0.23***	−0.17**	1			
4. Age	−0.10	−0.12*	0.04	1		
5. Household crowding index	0.07	−0.11*	−0.02	−0.09	1	
6. Body mass index	0.31***	0.19***	−0.24***	−0.11*	0.13*	1

* *p* < 0.05.

** *p* < 0.01.

*** *p* < 0.001.

### 3.2 Mediation Analysis

The mediation analysis results were adjusted for the following variables: sex, residence, age, HCI, and physical activity. BMI partially mediated the association between PSMU and body dysmorphic concerns (indirect effect: Beta = 0.03; BootSE = 0.02; and 95% BootCI 0.01; 0.07). Greater PSMU was significantly associated with higher BMI (a path: Beta = 0.18; SE = 0.07; *p* = 0.010; and 95% CI 0.04, 0.31). Higher BMI was also significantly associated with higher body dysmorphic concerns (b path: Beta = 0.20; SE = 0.05; *p* < 0.001; and 95% CI 0.10, 0.29). The total effect of PSMU on body dysmorphic concerns was significant (c path: Beta = 0.51, SE = 0.06, *p* < 0.001, and 95% CI 0.39, 0.63) and was directly associated with higher body dysmorphic concerns (direct effect, c′ path: Beta = 0.48, SE = 0.06, *p* < 0.001, and 95% CI 0.36, 0.60) (Table 3).

**TABLE 3:** Mediation effect of body mass index (BMI) between problematic social media use (PSMU) and body dysmorphic concern questionnaires (DCQ).

Effect/Path	Beta	SE/BootSE	*p*	95% CI
PSMU → BMI	0.18	0.07	0.010	0.04, 0.31
BMI → DCQ	0.20	0.05	<0.001	0.10, 0.29
Total effect: PSMU → DCQ	0.51	0.06	<0.001	0.39, 0.63
Direct effect: PSMU → DCQ	0.48	0.06	<0.001	0.36, 0.60
Indirect effect: PSMU → BMI → DCQ	0.03	0.02	—	BootCI 0.01, 0.07

Abbreviations: CI = confidence interval; SE = standard error.

## 4 Discussion

To the best of our knowledge, this study was the first to explore the mediating effect of BMI between body dysmorphic concerns and PSMU in a sample of young adults from the Lebanese population. However, when interpreting the mediation analysis, the cross‐sectional nature of our study allows us to reflect only statistical associations rather than causal pathways. Longitudinal studies are, therefore, needed to confirm the temporal sequence between PSMU, BMI, and body dysmorphic concerns. Overall, the present findings suggest that PSMU is associated with higher body dysmorphic concerns, both directly and indirectly through BMI, highlighting the complex interplay between behavioral, psychological, and physical factors.

First, higher PSMU appears to be associated with increased body dysmorphic concerns. In accordance with our findings, multiple studies [35, 50, 51] have suggested that PSMU can engender body dysmorphic concerns through multiple mechanisms, including self‐objectification and social comparison [18, 20]. A possible explanation is that social media platforms foster environments dominated by idealized body representations, leading individuals with higher PSMU to engage in frequent upward comparisons. In fact, it is well known that social media fosters an environment where objectification prevails, exposing individuals to unrealistic body standards that may lead them to fixate on minor or even nonexistent imperfections, thereby exacerbating dysmorphic concerns [21, 22]. Moreover, studies have shown that individuals engaging with fitness‐related content, particularly young adults, may develop body dysmorphic concerns, reflected in extreme behaviors, such as rigid exercise routines or restrictive dieting in response, to internalized body ideals [52]. This is particularly relevant in young adults, who are more vulnerable to body dissatisfaction and identity‐related pressures, further amplifying the impact of PSMU on body image disturbances [53].

Second, PSMU may also be linked to BMI, suggesting a potential behavioral pathway. In agreement with previous studies [29, 30], this relationship may reflect behavioral patterns, such as increased sedentary time, that have been reported among individuals with higher levels of social media use, which can reduce physical activity and contribute to higher BMI [29]. Additionally, emotional mechanisms may play a role, as PSMU has been associated with increased stress, anxiety, and depression, which, in turn, may promote maladaptive coping strategies, such as emotional eating. Conversely, other studies have reported an inverse association, suggesting that exposure to thin and idealized body images may lead some individuals to engage in restrictive eating behaviors in an attempt to lose weight [54]. These findings indicate that the relationship between PSMU and BMI is likely complex and influenced by individual differences in coping and internalization processes.

Third, BMI itself appears to be associated with body dysmorphic concerns, supporting its role as a partial mediator. In line with previous research [27, 55, 56], higher BMI may be associated with a more negative self‐perception and body dissatisfaction, increasing vulnerability to dysmorphic concerns. Studies have shown that individuals with higher BMI may internalize negative societal attitudes toward body image, leading to increased body‐related distress and self‐criticism [28, 56]. However, it is important to note that body dysmorphic concerns are not limited to individuals with higher BMI, as they may also occur across a wide range of body types [25]. Furthermore, although the indirect effect through BMI reached statistical significance, its magnitude was relatively small, indicating that BMI likely only partially explains the association between PSMU and body dysmorphic concerns. This highlights the likely involvement of additional psychological mechanisms, such as appearance‐based social comparison and the internalization of sociocultural beauty ideals promoted on social media, which may also contribute to this relationship [20, 51].

To the best of our knowledge, no research has been performed yet in Lebanon to explore the association between PSMU, BMI, and body dysmorphic concerns. Thus, the present study contributes novel evidence within this cultural context. From a practical perspective, these findings suggest that interventions targeting PSMU should not only focus on reducing screen time but also address cognitive and emotional processes, such as comparison tendencies and body image internalization. Higher PSMU was associated with higher BMI and greater body dysmorphic concerns in our sample. These associations may coexist with behavioral and psychological factors, such as sedentary behavior, emotional eating, and body image dissatisfaction; however, the temporal relationships among these variables cannot be determined from the present study [20, 57].

### 4.1 Clinical Implications

Given the results of our study, the need for inclusive support strategies that addresses social media use, BMI, and body dysmorphic concerns was highlighted. In fact, the mediating effect of BMI on the association between PSMU and body dysmorphic concerns among the young adult population in Lebanon underlines a multifaced clinical challenge engrained in self‐image, cultural body ideals, and social media influence. Young Lebanese adults may experience heightened body dissatisfaction when exposed to idealized body images on social media, as they often aspire to meet demanding standards of physical appearance. High BMI may be associated with greater body‐related concerns, nurturing a greater propensity to develop body dysmorphic concerns, considering that individuals might compare themselves to unrealistic representations online. In this context, strategies that address body image perceptions, patterns of social media use, and weight‐related concerns may represent relevant components of mental health promotion among university populations. Early identification of body dysmorphic concerns, alongside increased awareness of potentially harmful appearance‐related content on social media, may also help support healthier self‐perceptions. Besides, in a cultural background like Lebanon, where societal expectations around appearance are influential, underscoring a culturally tailored framework is fundamental. Combining cognitive and physical health interventions, clinicians can help individuals foster a more balanced, authentic, and healthy self‐image, while developing resilience against social media stereotypes and pressures.

Nevertheless, it is important to note that the indirect association observed through BMI was relatively small in magnitude and should therefore be interpreted with caution. Although BMI was statistically associated with the relationship between PSMU and body dysmorphic concerns, it is likely only one of several factors involved. Other psychological and sociocultural processes, including appearance‐based social comparison, self‐objectification, internalization of appearance ideals, and body dissatisfaction, may also contribute to these associations. Consequently, the present findings should be viewed as highlighting a potentially relevant correlate rather than suggesting a predominant explanatory role for BMI.

### 4.2 Limitations and Strengths

In order to preserve the scientific validity of our study, there might be many limitations worth being highlighted. Primarily, the cross‐sectional design of our study restrains our capability to draw causality and establish temporal relationships between our three variables. Although mediation analysis can illustrate potential explanatory pathways, it cannot confirm causal mechanisms.

Moreover, self‐reported questionnaires may present risks of social desirability bias, along with information bias, knowing that we do not have control over how responders comprehend the questions before answering them, without forgetting the predominance, in our study, of single over married individuals, which could possibly influence the results obtained. Additionally, BMI was calculated using self‐reported height and weight, which may introduce reporting bias, as individuals tend to underestimate their weight and overestimate their height. Such bias may have influenced the precision of the BMI estimates. Self‐reported BMI is likely to be underestimated, which may attenuate the strength of the association between BMI and body dysmorphic concerns. In addition, body dysmorphic concerns were assessed using the DCQ, which is a screening instrument and does not provide a clinical diagnosis of body dysmorphic disorder. Therefore, our findings reflect subclinical dysmorphic concerns rather than confirmed psychiatric diagnoses, which may limit clinical interpretation. The sampling method by “convenience” may also impose a selection bias due to online recruitment that might have led to an overrepresentation of individuals with higher social media use, potentially overestimating the association between PSMU and body dysmorphic concerns. Residual bias might also be present as we could not be able to include all variables related to body dysmorphic concerns in our questionnaire. According to the best of our knowledge, to date, no study has investigated the mediating effect of BMI between PSMU and body dysmorphic concerns in the Lebanese young adult population, which emphasizes the originality of our work despite the literature challenges.

## 5 Conclusion

In summary, being the first of its kind to examine this multifaced subject, our study provided evidence of the correlation between PSMU and body dysmorphic concerns, with BMI statistically accounting for an indirect mediating effect in this association, within the Lebanese young adult population. These findings could pave the way for implementing a multilayered strategy aimed at helping individuals nurture a more balanced, authentic, and healthy self‐perception, while building resilience to withstand social media pressures. Yet considering the existent limitations, further studies allowing the establishment of a cause‐and‐effect relationship could also be launched on this complex subject while identifying other possible mediators of this association for a better understanding of the dynamic between the variables. Moreover, studies conducted in different populations and different settings could assess if our results are local or generalizable.

## Author Contributions

Feten Fekih‐Romdhane, Souheil Hallit, and Marie Hokayem designed the study. Simona Mahfouz collected the data. Carla Hokayem drafted the manuscript. Michelle Abi Karam and Nada Akiki helped with the writing. Souheil Hallit carried out the analysis and interpreted the results. Sahar Obeid revised the article for intellectual content. All authors reviewed the final manuscript and gave their consent.

## Funding

The authors have nothing to report.

## Ethics Statement

This study received an approval from the ethical committee of the Holy Spirit University of Kaslik (USEK) (research ethics certificate number: HCR/EC 2024‐031). A written informed consent form was mandatory to be filled in order to voluntarily and freely complete the survey. All methods were performed in accordance with the relevant guidelines and regulations (in accordance with the Declaration of Helsinki).

## Consent

The authors have nothing to report.

## Conflicts of Interest

The authors declare no conflicts of interest.

## Data Availability

The data that support the findings of this study are available from the corresponding author but restrictions apply to the availability of these data, which were used under license for the current study and so are not publicly available. Data are, however, available from the authors upon reasonable request and with permission of the ethics committee.
